# MisPred: a resource for identification of erroneous protein sequences in public databases

**DOI:** 10.1093/database/bat053

**Published:** 2013-07-17

**Authors:** Alinda Nagy, László Patthy

**Affiliations:** Institute of Enzymology, Research Centre for Natural Sciences, Hungarian Academy of Sciences, H-1113 Budapest, Hungary

## Abstract

Correct prediction of the structure of protein-coding genes of higher eukaryotes is still a difficult task; therefore, public databases are heavily contaminated with mispredicted sequences. The high rate of misprediction has serious consequences because it significantly affects the conclusions that may be drawn from genome-scale sequence analyses of eukaryotic genomes. Here we present the MisPred database and computational pipeline that provide efficient means for the identification of erroneous sequences in public databases. The MisPred database contains a collection of abnormal, incomplete and mispredicted protein sequences from 19 metazoan species identified as erroneous by MisPred quality control tools in the UniProtKB/Swiss-Prot, UniProtKB/TrEMBL, NCBI/RefSeq and EnsEMBL databases. Major releases of the database are automatically generated and updated regularly. The database (http://www.mispred.com) is easily accessible through a simple web interface coupled to a powerful query engine and a standard web service. The content is completely or partially downloadable in a variety of formats.

**Database URL:** http://www.mispred.com

## Introduction

Computational gene prediction is one of the key issues in bioinformatics, as emphasis moved from large-scale sequencing of genomes to knowledge extraction from genome sequences. In the past two decades, several hundred programs have been designed for the identification of genes (1). In the case of prokaryotic genomes, prediction of the structure of protein-coding genes is reliable, but in the case of intron-rich genomes of higher eukaryotes, prediction of the correct structure of protein-coding genes remains a difficult task.

Recent analyses have shown that the exact genomic structure of protein-coding genes of higher eukaryotes is correctly predicted for only ∼60% of the genes (2, 3). An increasing number of studies confirm that misprediction is a far more serious problem than previously thought (4) and that contamination of public databases with erroneous sequences may significantly distort the results of genome-scale evolutionary analyses (5–8). The main objective of our MisPred project is to identify erroneous (abnormal, incomplete and mispredicted) protein sequences in public databases to improve the quality of these databases. The rationale of the MisPred approach is that the structure of a protein-coding gene is likely to be mispredicted if some of the features of the predicted protein conflict with our current knowledge about proteins. The current version of the MisPred computational pipeline uses 11 distinct tools to identify erroneous sequences; the MisPred database contains a total of 80 890 erroneous sequences identified in 19 metazoan species.

## Database generation

We have designed the MisPred pipeline to identify abnormal, incomplete and mispredicted proteins primarily from metazoan genomes. The MisPred pipeline takes a protein sequence as input and checks if its features conflict with our current knowledge about proteins and protein-coding genes. For each sequence, the following MisPred tools are used for quality control:

Tool 1. Conflict between the presence of extracellular protein domain(s) in a protein and the absence of appropriate sequence signals that could direct the extracellular domain(s) into the extracellular space. Rationale: proteins containing domains that occur exclusively in the extracellular space (e.g. in secreted extracellular proteins or in the extracellular parts of type I, type II, type III single-pass transmembrane proteins or multispanning transmembrane proteins) must have a secretory signal peptide, signal anchor or transmembrane helix(ces).
Tool 2. Conflict between the presence of extracellular and cytoplasmic domains in a protein and the absence of transmembrane helix(ces). Rationale: extracellular protein domains and cytoplasmic protein domains can co-occur in multidomain proteins only if they also contain transmembrane helix(ces) that pass through the membrane.
Tool 3. Co-occurrence of extracellular and nuclear domains in a protein. Rationale: nuclear protein domains do not co-occur with extracellular protein domains in multidomain proteins (9).
Tool 4. Domain size deviation. Rationale: the number of amino acid residues in a domain family usually fall in a relatively narrow range.
Tool 5. Interchromosomal chimeric proteins. Rationale: a protein is encoded by exons located on a single chromosome.
Tool 6. Conflict between the presence of secretory signal peptide and cytoplasmic protein domains in a protein and the absence of transmembrane segments. Rationale: secretory signal peptide and cytoplasmic protein domains can co-occur in proteins only if they also contain transmembrane helix(ces) that pass through the membrane.
Tool 7. Conflict between the presence of glycosylphosphatidylinositol (GPI) anchor in a protein and the absence of secretory signal peptide. Rationale: a protein that is to be attached to the outer cell membrane via a C-terminal GPI anchor contains a secretory signal peptide that directs it to the extracellular space.
Tool 8. Co-occurrence of GPI anchor and cytoplasmic protein domains in a protein. Rationale: in the case of GPI-anchored proteins, the whole protein resides in the extracellular space; therefore, they do not contain cytoplasmic protein domains.
Tool 9. Co-occurrence of GPI anchor and nuclear protein domains in a protein. Rationale: in the case of GPI-anchored proteins, the whole protein resides in the extracellular space; therefore, they do not contain nuclear protein domains.
Tool 10. Co-occurrence of GPI anchor and transmembrane segments in a protein. Rationale: in the case of GPI-anchored proteins, the whole protein resides in the extracellular space; therefore, they do not contain transmembrane helices.
Tool 11. Domain architecture deviation. Rationale: changes in domain architecture of proteins are relatively rare evolutionary events (whereas the error rate in gene prediction is relatively high); therefore, if we find a hypothetical protein with a ‘novel’ domain architecture, then this is more likely to reflect an error in gene prediction than true innovation (10). (It must be pointed out that MisPred tool 11 is not yet available in searches on the MisPred website and that data generated with this tool are not yet deposited in the MisPred database. This tool will be released in the next update of MisPred.)

The different MisPred tools combine the results obtained by analysis of the sequences for the presence of protein domains and sequence features (signal peptide, signal anchor, transmembrane helix, GPI anchor). Pfam-A domains are identified using the Pfam database and the HMMER program (11, 12); CDD domains are identified by reversed position specific blast against the Conserved Domain Database using Pfam-derived position-specific scoring matrices (13); secretory signal peptides are identified by Predisi and SignalP (14, 15); transmembrane helices and signal anchor sequences are identified by TMHMM and Phobius (16, 17); and GPI anchors are identified by DGPI (18). The lists of obligatory extracellular, cytoplasmic and nuclear Pfam-A domains were defined as described previously (4) and are found in Tables 1–3 at http://www.mispred.com/table1to3. The list of Pfam-A domain families suitable for the study of domain integrity is shown in Table 4 at http://www.mispred.com/table4. Interchromosomal protein chimeras are identified based on the results of BLAST-like alignment tool (BLAT) search of the appropriate genomes (19).

We have analyzed protein sequences of 19 metazoan species deposited in the UniProtKB/Swiss-Prot, UniProtKB/TrEMBL (20), NCBI/RefSeq (21) and EnsEMBL (22) databases. Protein sequences were downloaded from the given website, and each protein sequence was analyzed with all of the MisPred tools. Protein sequences identified as erroneous by the MisPred tools are stored in the MisPred database along with their MisPred annotation.

A more detailed description of the constituents, tool logic and performance of the various MisPred tools, as well as the datasets analyzed, are found in online supplementary material.

## Database content

**Figure 1.**
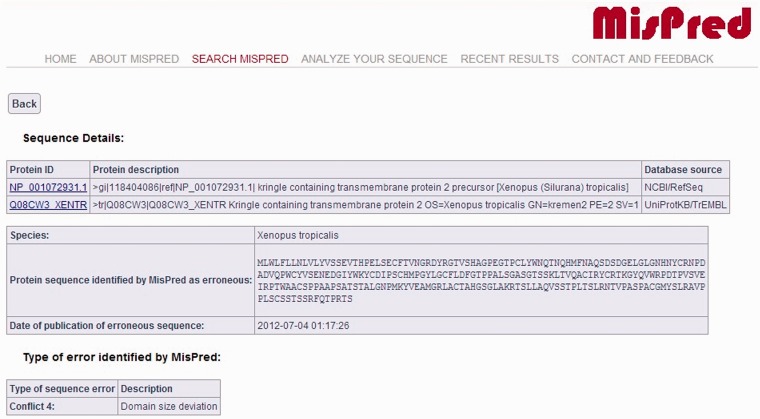
MisPred annotation of an erroneous protein sequence. The figure shows the entry for a protein sequence of *X. tropicalis* deposited in NCBI/RefSeq database with the protein ID: NP_001072931.1 and in the UniProtKB/TrEMBL database with the protein ID: Q08CW3_XENTR. The protein was identified as erroneous by MisPred tool 4 (domain size deviation) because it contains only a fragment of a domain (Pfam-A domain PF01822, WSC).

The current version of the MisPred database contains 80 890 abnormal, incomplete and mispredicted proteins from 19 metazoan species (*Homo sapiens*, *Mus musculus*, *Rattus norvegicus*, *Monodelphis domestica*, *Gallus gallus*, *Xenopus tropicalis*, *Danio rerio*, *Fugu rubripes*, *Ciona intestinalis*, *Branchiostoma floridae*, *Strongylocentrotus purpuratus, Drosophila melanogaster*, *Drosophila simulans*, *Drosophila pseudoobscura*, *Caenorhabditis elegans*, *Caenorhabditis briggsae*, *Hydra magnipapillata*, *Nematostella vectensis*, *Trichoplax adhaerens*)*.* For each protein sequence, the protein ID, the protein description, the database source, the species name and the type of sequence error(s) identified by MisPred are provided. A typical example of an erroneous sequence in a public database is shown in Figure 1.

It should be noted that if the same erroneous sequence was deposited multiple times in the same or different databases, several protein IDs and database sources are listed. MisPred entries also display the actual protein sequence analysed by MisPred, together with the date of analysis by MisPred. The justification for the inclusion of this information in the MisPred database is that some databases retain the original protein ID when the curators of the database replace the erroneous sequence by a corrected sequence.

## Database statistics

The current version of MisPred contains 80 890 abnormal, incomplete and mispredicted proteins. Of these protein sequences, 2245 were found in UniProtKB/Swiss-Prot, 65 786 in UniProtKB/TrEMBL, 24 996 in NCBI/RefSeq and 34 050 in EnsEMBL. Tables 1 and 2 summarize the percentages of mispredicted sequences in various databases and in 19 metazoan species. Further details of the statistics of erroneous sequences of different databases (organism, type of sequence errors, etc.) are found in the ‘Statistics’ that may be displayed via a link on the ‘ABOUT MISPRED’ page of the website (http://www.mispred.com).

**Table 1.** Percentage of mispredicted sequences in various databases

Database	Number of proteins	Identified as suspicious by MisPred	Percentage (%)
UniProtKB/SwissProt (release 2012_05, May 2012)	59 000	2245	3.81
UniProtKB/TrEMBL (release 2012_05, May 2012)	598 362	65 786	10.99
EnsEMBL (release 67, May 2012)	392 818	34 050	8.67
NCBI/RefSeq (May 2012)	374 046	24 996	6.68

**Table 2.** Percentage of mispredicted sequences in 19 metazoan species

Species	Number of proteins	Identified as suspicious by MisPred	Percentage (%)
*Homo sapiens*			
UniProtKB/SwissProt	20 215	762	3.77
UniProtKB/TrEMBL	101 629	22 790	22.42
EnsEMBL	83 139	10 798	12.99
NCBI/RefSeq	23 125	1183	5.12
*Mus musculus*			
UniProtKB/SwissProt	16 526	588	3.56
UniProtKB/TrEMBL	61 249	6609	10.79
EnsEMBL	50 702	4471	8.82
NCBI/RefSeq	26 251	1096	4.18
*Rattus norvegicus*			
UniProtKB/SwissProt	7750	251	3.24
UniProtKB/TrEMBL	33 859	2637	7.79
EnsEMBL	32 780	2042	6.23
NCBI/RefSeq	25 304	1212	4.79
*Gallus gallus*			
UniProtKB/SwissProt	2244	100	4.46
UniProtKB/TrEMBL	26 611	2649	9.95
EnsEMBL	21 866	1874	8.57
NCBI/RefSeq	17 365	990	5.70
*Monodelphis domestica*			
UniProtKB/SwissProt	43	2	4.65
UniProtKB/TrEMBL	32 893	2452	7.45
EnsEMBL	32 422	2359	7.28
NCBI/RefSeq	18 979	996	5.25
*Xenopus tropicalis*			
UniProtKB/SwissProt	1655	56	3.38
UniProtKB/TrEMBL	28 865	1898	6.58
EnsEMBL	22 579	1522	6.74
NCBI/RefSeq	22 515	1288	5.72
*Danio rerio*			
UniProtKB/SwissProt	2821	159	5.64
UniProtKB/TrEMBL	53 846	5307	9.86
EnsEMBL	39 423	3662	9.29
NCBI/RefSeq	26 263	2094	7.97
*Fugu rubripes*			
UniProtKB/SwissProt	173	12	6.94
UniProtKB/TrEMBL	48 816	4873	9.98
EnsEMBL	47 728	3999	8.38
NCBI/RefSeq	442	15	3.39
*Ciona intestinalis*			
UniProtKB/TrEMBL	19 010	1523	8.01
EnsEMBL	17 281	1377	7.97
NCBI/RefSeq	13 825	837	6.05
*Branchiostoma floridae*			
UniProtKB/SwissProt	55	1	1.82
UniProtKB/TrEMBL	29 164	3040	10.42
NCBI/RefSeq	29 226	3042	10.41
*Strongylocentrotus purpuratus*			
UniProtKB/SwissProt	109	7	6.42
UniProtKB/TrEMBL	29 430	3189	10.84
NCBI/RefSeq	24 414	2804	11.49
*Drosophila melanogaster*			
UniProtKB/SwissProt	3152	136	4.31
UniProtKB/TrEMBL	33 786	2103	6.22
EnsEMBL	19 460	853	4.38
NCBI/RefSeq	19 577	860	4.39
*Drosophila simulans*			
UniProtKB/SwissProt	199	8	4.02
UniProtKB/TrEMBL	18 624	1390	7.46
NCBI/RefSeq	15 359	954	6.21
*Drosophila pseudoobscura*			
UniProtKB/TrEMBL	495	34	6.87
NCBI/RefSeq	15 995	824	5.15
*Caenorhabditis elegans*			
UniProtKB/SwissProt	3354	140	4.17
UniProtKB/TrEMBL	22 309	971	4.35
EnsEMBL	25 438	1093	4.30
NCBI/RefSeq	23 229	1056	4.55
*Caenorhabditis briggsae*			
UniProtKB/SwissProt	566	17	3.00
UniProtKB/TrEMBL	21 341	1007	4.72
NCBI/RefSeq	19 222	986	5.13
*Hydra megnipapillata*			
UniProtKB/TrEMBL	157	7	4.46
NCBI/RefSeq	17 002	1467	8.63
*Nematostella vectensis*			
UniProtKB/SwissProt	115	5	4.35
UniProtKB/TrEMBL	24 717	2493	10.09
NCBI/RefSeq	24 430	2483	10.16
*Trichoplax adhaerens*			
UniProtKB/SwissProt	23	1	4.35
UniProtKB/TrEMBL	11 561	814	7.04
NCBI/RefSeq	11 523	809	7.02

## Database implementation

The database is built on an Apache HTTP Server 2.2.6 with Oracle Database 11g Server. The front-end was developed using play! 1.2.4 (http://www.playframework.org) framework with HTML and JAVA script, and the back-end was developed using Oracle Database 11g Server, a relational database management system. All common gateway interface and database interfacing scripts were written in Java programming language.

## Web interface

The MisPred web interface is designed to explain the principles of the MisPred approach (web page ABOUT MISPRED) and to allow the user to rapidly query the complete database (web page SEARCH MISPRED) or to use the various MisPred tools to check sequences for potential errors (web page ANALYZE YOUR SEQUENCE).

### Search tools

MisPred provides two search options on the ‘SEARCH MISPRED’ page: the simple and the advanced search options. The simple search option allows users to query any field of the database entries (protein ID of the source database, protein description, database source, species name, type of sequence error identified by MisPred). Under the advanced search option, users can combine queries of the different fields using the AND, OR and NOT operators.

Upon initiating the search, the IDs of all protein sequences matching the criteria of the search are displayed. In the case of protein sequences that are present in several databases, all protein IDs of the sequence are displayed in one line. For each protein sequence retrieved (see Figure 1), a detailed result page is displayed (via a link of the protein ID) providing basic information about the protein sequence, including protein ID(s), protein description(s), database source(s), species name, amino acid sequence of the protein at the time of MisPred analysis, the date of MisPred analysis and the type of sequence error(s) identified by MisPred. Links to the source databases are also provided to help the user retrieve supplementary information about the protein. Selected sequences may be downloaded in a variety of formats (XML, EXCEL, FASTA, LIST).

### Sequence analysis tools

Users can analyse protein sequences for possible sequence errors on the ‘ANALYZE YOUR SEQUENCE’ page using the MisPred quality control tools. The results of the analysis are accessible in two different ways: without registration the results are available via a link for 72 hours; registered users can access their results on the ‘Recent Results’ page for 20 days.

**Figure 2.**
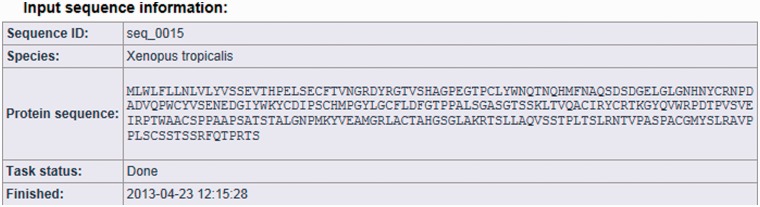
MisPred analysis of a protein sequence for potential sequence errors. The sequence shown in Figure 1 was analysed with the various MisPred tools. This figure shows basic information about the input protein sequence (automatically generated sequence ID, species name, protein sequence, task status and date and time of the completion of the analysis).

**Figure 3.**
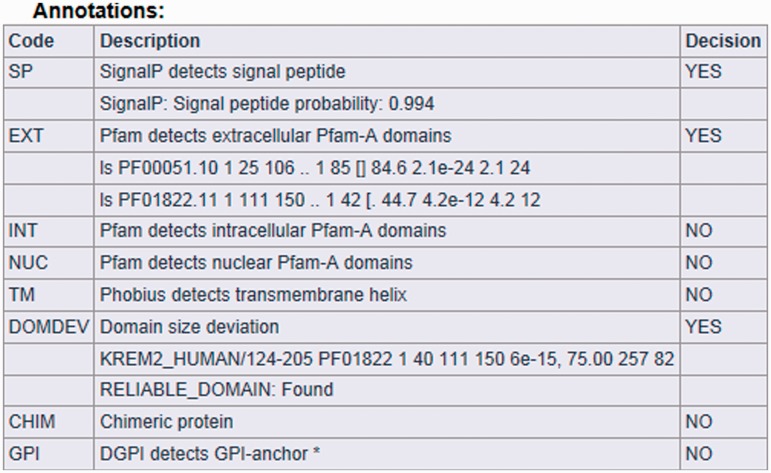
MisPred analysis of a protein sequence for potential sequence errors. The sequence shown in Figure 1 was analysed with the various MisPred tools. The figure shows the primary conclusions based on the analyses for signal peptide, Pfam-A domains, transmembrane helix, GPI anchor, domain-size integrity and chromosomal localization of the exons encoding the protein. In the rows showing the Pfam-A domains present in this protein, the different characters represent the output of the HMMscan program. For example, in the first row, the characters (from left to right) indicate the Model used (ls), the domain type identified (PF00051.10), the number of copies of this domain type in this protein (1), the first and last residues of the domain, defined by residue numbering of this protein (25 106), the first and last residues of the HMM of this domain type that align with PF00051 of this protein (1 85), the score of the match (84.6) and the *E*-value of the match (2.1 e-24). Note that these analyses revealed that the protein is a secreted extracellular protein that contains a secretory signal peptide and two types of extracellular domains. In harmony with the extracellular localization of the protein, it does not contain intracellular signaling domains, nuclear domains or transmembrane helices. However, the protein is erroneous in as much as one of its extracellular protein domains, the Pfam-A domain PF01822 (WSC-domain) is truncated, an error that is detected by MisPred tool 4 (domain-size deviation).

**Figure 4.**
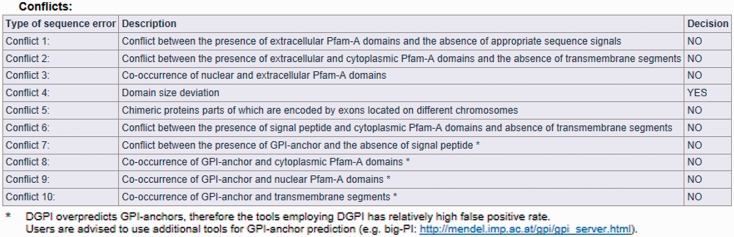
MisPred analysis of a protein sequence for potential sequence errors. The sequence shown in Figure 1 was analysed with the various MisPred tools. This figure summarizes the conclusions: the sequence violates only one of the MisPred rules: the size of one of its domains deviates significantly from the size typical of the given domain family. Note that conflict 11 is missing from the type of sequence errors, as MisPred tool 11 is not yet available in searches on the MisPred website. This tool will be released in the next update of MisPred.

The result page is divided into three parts (see Figures 2, 3 and 4). The first section displays basic information about the protein sequence submitted for analysis (automatically generated sequence ID, species name, protein sequence, task status and date and time of the completion of the analysis). The second section shows the sequence annotations obtained by MisPred analysis (presence or absence of signal peptide, transmembrane helices, etc.). The third section summarizes the conclusion of the MisPred analysis (lists the type(s) of sequence error(s) identified by the MisPred tools).

## Conclusions and future perspectives

The principle of the MisPred approach is that a protein sequence is likely to be erroneous if some of its features conflict with our current knowledge about proteins. The current version of MisPred uses a variety of tools that were optimized for the analysis of proteins of Metazoa, but because the validity of this basic principle is not restricted to metazoan proteins, in our future work we will adapt the MisPred tools for analysis of proteins of other eukaryotes. In the future, we plan to update the sequence content of the MisPred database twice a year.

We believe that MisPred will prove to be an important resource for the quality control of protein sequences and will contribute to a significant improvement in the quality of public sequence databases.

## Supplementary Data

Supplementary data are available at *Database* Online.

## Funding

National Office for Research and Technology of Hungary (TECH_09_A1-FixPred9) and the Hungarian Scientific Research Fund (OTKA 101201). Funding for open access charge: Hungarian Scientific Research Fund (OTKA 101201).

*Conflict of interest*. None declared.

## Supplementary Material

Supplementary Data
