# Supplementary material for: MisPred: a resource for identification of erroneous protein sequences in public databases
Source: Database (Oxford). 2013 Jul 17;2013:bat053. doi: 10.1093/database/bat053 (PMC3713709; doi:10.1093/database/bat053)
Supplement: Supplementary Data [file supp_2013_bat053_index.html]

MisPred: a resource for identification of erroneous protein sequences in public databases — Supplementary Data 

# MisPred: a resource for identification of erroneous protein sequences in public databases

## 

files

**Files in this Data Supplement:**

- Supplementary Data - pdf file
