# Supplementary material for: MisPred: a resource for identification of erroneous protein sequences in public databases
Source: Database (Oxford). 2013 Jul 17;2013:bat053. doi: 10.1093/database/bat053 (PMC3713709; doi:10.1093/database/bat053)
Supplement: Supplementary Data [file supp_bat053_Supplementary_file1.pdf]

MisPred: a resource for identification of erroneous protein sequences in public databases

Alinda Nagy and László Patthy
Institute of Enzymology, Research Centre for Natural Sciences, Hungarian Academy of Sciences, H-1113 Budapest, Hungary

Description of MisPred tools

1. Constituents of MisPred tools

1.1. Lists of extracellular, cytoplasmic and nuclear Pfam-A domains

Recent studies have revealed that there is a strong correlation between the domain-composition of proteins and their subcellular location: some domains are restricted to proteins targeted to the extracellular space, others occur only in proteins present in the cytoplasmic space, while others are restricted to proteins of the nucleus. Transmembrane multidomain proteins are special in the sense that extracellular and cytoplasmic domains can legitimately co-occur in a single protein (Tordai *et al*., 2005). Accordingly, the presence of extracellular, cytoplasmic or nuclear domains in a protein may be used to predict its subcellular localization, independent of the detection of sorting signals.

Since domains are most likely to co-occur in multidomain proteins if they belong to the same localization-category, analysis of domain co-occurrence networks is useful for the systematic assignment of domains to different subcellular compartments (Tordai *et al*., 2005).

Our domain co-occurrance analyses of Metazoan UniProtKB entries have identified 166 extracellular, 115 cytoplasmic and 126 nuclear Pfam-A domain families as being restricted to the respective subcellular compartment, the majority of which are also identified as such in the SMART database http://smart.embl-heidelberg.de/ (see http://www.mispred.com/table1to3).

In our MisPred analyses only these extracellular or cytoplasmic or nuclear domain families were used to predict subcellular localization. Pfam-A domains that are known not to be restricted to a particular cellular compartment, such as immunoglobulin domains, fibronectin type III domains, von Willebrand factor type A domains (i.e. domains that are 'multilocale'), are not reliable predictors of subcellular localization and thus they were not utilized in these analyses.

1.2. Programs used for the identification of Pfam-A domains

The programs of the HMMER 3.0 software package were used to detect Pfam-A domains in proteins (Finn *et al*. 2011; Punta *et al.* 2012). Pfam-A domains were detected by searching the HMM databases with the hmmscan program using 1e-6 as per-domain E-value threshold. The results were filtered for overlapping domain matches and only the match with the lowest E-value was accepted.

The HMMER software package was obtained from http://hmmer.janelia.org/. HMM databases of Pfam-A domains were created by retrieving the HMMs of the domains from Pfam HMM libraries downloaded from ftp://ftp.sanger.ac.uk/pub/databases/Pfam/. The local Pfam HMM libraries will be updated twice a year.

1.3. Programs used for the identification of secretory signal peptides

Proteins were analyzed with the PrediSi program (Hiller *et al*. 2004), the SignalP 3.0 program (Bendtsen *et al*. 2004) to identify the presence of eukaryotic signal peptide sequences.

The PrediSi program was downloaded from http://www.predisi.de/, the SignalP program was downloaded from http://www.cbs.dtu.dk/cgi-bin/sw_request?signalp+3.0.

1.4. Programs used for the identification of transmembrane helices

Proteins were analyzed with the TMHMM 2.0 program (Krogh *et al*. 2001) and the Phobius program (Käll *et al*. 2007) to detect the presence of transmembrane helices.

The TMHMM program was obtained from http://www.cbs.dtu.dk/services/TMHMM/. The Phobius program was obtained from http://software.sbc.su.se/cgi-bin/request.cgi?project=phobius.

1.5. Program used for the identification of GPI-anchors

Protein sequences were analyzed with the DGPI program (Kronegg and Buloz, 1999) to identify GPI-anchors.

The DGPI program was obtained from http://dgpi.pathbot.com/.

1.6. Program used for the chromosomal localization of exons encoding a protein

The protein sequences were matched to the genome of the given species using the BLAT program (Kent, 2002).

The BLAT program was obtained from http://genome-test.cse.ucsc.edu/~kent/exe/.

2. MisPred tool logic

Tool 1.

Conflict between the presence of extracellular protein domain(s) in a protein and the absence of appropriate sequence signals that could direct the extracellular domain(s) into the extracellular space.

Proteins found to contain extracellular Pfam-A domains were analyzed by the PrediSi program (using 0.3 as threshold) and the SignalP 3.0 program to identify the presence of eukaryotic signal peptide sequences and by the TMHMM 2.0 program and the Phobius program to detect the presence of transmembrane helices.

Protein sequences containing extracellular domains but lacking a signal peptide or a transmembrane helix were identified as suspicious.

Tool 2.

Conflict between the presence of extracellular and cytoplasmic domains in a protein and the absence of transmembrane helix(ces).

Proteins found to contain both extracellular and cytoplasmic Pfam-A domains were analyzed by the TMHMM 2.0 and the Phobius programs to detect transmembrane helices.

Sequences containing both extra- and cytoplasmic domains (i.e. putative transmembrane proteins) but lacking a transmembrane helix were identified as suspicious.

Tool 3.

Co-occurrence of extracellular and nuclear domains in a protein.

Protein found to contain both extracellular and nuclear Pfam-A domains were identified as suspicious.

Tool 4.

Domain size deviation.

The rationale of this tool is that the number of amino acid residues in a Pfam-A domain-family usually falls in a relatively narrow range. MisPred tool 4 uses only Pfam-A domain families that have a well-defined, conserved sequence length range: their members in the high quality Swiss-Prot database do not deviate from the average size by more than 2 standard deviation (SD) values. Based on these criteria, about 90% of the Pfam-A domain families present in Swiss-Prot proteins proved to be suitable for the study of domain integrity. The lists of Pfam-A domain families suitable for the study of domain integrity are shown in http://www.mispred.com/table4.

We created local databases of human, vertebrate and metazoa+fungi Swiss-Prot domain sequences belonging to the Pfam-A families suitable for the study of domain integrity and ran a blastp search with the protein queries against the appropriate Swiss-Prot domain sequences.

MisPred tool 4 selects those partial domain matches which share over 60% identity with the query sequence, with an E-value < 1e-5 but they differ in length by at least 40%. Protein sequences containing domains with such deviant lengths were identified as suspicious.

Tool 5.

Interchromosomal chimeric proteins.

MisPred tool 5 matches protein sequences to the genome of the given species using the BLAT program and lists matches with >95% identity over ≥ 15 amino acid residues in length. In the case of overlapping matches (if the overlap was >5 residues) the tool selects the longest match. To eliminate problems encountered with genes originating from the mitochondrial genome MisPred tool 5 uses an additional BLAT search and discards those entries which gave >90% match with the mitochondrial genome over more than 90% of their length.

MisPred tool 5 identifies proteins as interchromosomal chimeras if two or more of their segments are encoded on different chromosomes.

Tool 6.

Conflict between the presence of secretory signal peptide and cytoplasmic protein domains in a protein and the absence of transmembrane segments.

Proteins found to contain cytoplasmic Pfam-A domains were analyzed by the PrediSi and the SignalP 3.0 programs to identify the presence of eukaryotic signal peptide sequences and by the TMHMM 2.0 and the Phobius programs to detect the presence of transmembrane helices.

Protein sequences containing cytoplasmic domains and signal peptide sequences (i.e. putative transmembrane proteins) but lacking a transmembrane helix were identified as suspicious.

Tool 7.

Conflict between the presence of GPI-anchor in a protein and the absence of secretory signal peptide.

Protein sequences were analyzed with the DGPI program to identify GPI-anchors. Proteins predicted to contain a GPI-anchor were analyzed with the PrediSi and the SignalP 3.0 programs to identify the presence of eukaryotic signal peptide sequences.

Proteins that contain a GPI-anchor but lack a secretory signal peptide were identified as suspicious.

Tool 8.

Co-occurrence of GPI-anchor and cytoplasmic protein domains in a protein.

Protein sequences were analyzed with the DGPI program to identify GPI-anchors. Proteins predicted to contain a GPI-anchor were analyzed with the hmmscan program to identify the presence of cytoplasmic Pfam-A domains.

Protein sequences containing both GPI-anchor and cytoplasmic domains are identified as suspicious.

Tool 9.

Co-occurrence of GPI-anchor and nuclear protein domains in a protein.

Protein sequences were analyzed with the DGPI program to identify GPI-anchors. Proteins predicted to contain a GPI-anchor were analyzed with the hmmscan program to identify the presence of nuclear Pfam-A domains.

Protein sequences containing both GPI-anchor and nuclear domains are identified as suspicious.

Tool 10.

Co-occurrence of GPI-anchor and transmembrane segments in a protein.

Protein sequences were analyzed with the DGPI program to identify GPI-anchors. Proteins predicted to contain a GPI-anchor were analyzed with the TMHMM 2.0 and Phobius programs to identify the presence of transmembrane segments.

Protein sequences containing both GPI-anchor and transmembrane segments are identified as suspicious.

Tool 11.

Domain architecture deviation.

The rationale of this tool is that changes in domain architecture of proteins are relatively rare evolutionary events (whereas the error rate in gene prediction is relatively high), therefore if we find a protein whose domain architecture differs from those of its orthologs then this is more likely to reflect an error in gene prediction than true change in domain architecture.

MisPred tool 11 searches protein sequences for the presence of domains using RPS-BLAST against the Conserved Domain Database using Pfam-derived position-specific scoring matrices (Marchler-Bauer *et al*., 2013). The tool records domain hits with an e-value of <1e-5, eliminates overlapping hits and determines the domain architecture (DA, linear sequence of domains) at four different e-value cut-offs of domain hits: <1e-2, <1e-3, <1e-4 and <1e-5. The tool compares the DA with those of orthologs and in the case of DA difference (at all cut-off values) it recalculates their DA using the programs of the HMMER

3.0 software package and the Pfam HMM libraries at four different e-value cut-offs: <1e-2, <1e-3, <1e-4 and <1e-5.

MisPred tool 11 identifies protein sequences that differ in domain architecture (at any single cut-off value) from those of its orthologs as suspicious.

3. Specificity and sensitivity of MisPred tools

We have performed extensive analyses to optimize specificity and sensitivity of the various MisPred tools. False positive rates of the tools were monitored on high quality datasets (e.g. Swiss-Prot database) that are practically free of erroneous sequences. False negative rates of the tools were monitored on datasets of erroneous sequences generated artificially from correct Swiss-Prot sequences.

3.1. False positive rates and specificity

To calculate the false positive rate (α) and specificity ($1-\alpha$) of MisPred tools from the equation $\alpha = FP/(FP+TN)$, we have determined the number of false positives (FP) and true negatives (TN) from the results obtained by application of MisPred tools to high quality Swiss-Prot entries.

Considering that Swiss-Prot is a very clean database, in these calculations FP equaled the number of Swiss-Prot entries that were identified with the given method as suspicious (although they do not violate the given dogma), whereas the entries not identified by MisPred as suspicious were assumed to be true negatives (TN), i.e. they do not violate the given dogma.

To monitor false positive rate of MisPred tool 11 we have randomly selected 500 pairs of orthologous human, pongo, rat, mouse, chick, frog, zebrafish, worm and fly Swiss-Prot sequences from the list of orthologs and retained only pairs that align over their entire length and had identical domain architecture as evidenced by Swiss-Prot annotation.

The false positive rates of the MisPred routines 1-6 and 11 were calculated to be $\leq$ 0.015, i.e. their specificity is very high ($\geq$ 0,985). The high specificity of these tools reflects the fact that the constituent programs have high specificity and that these MisPred tools are based on generally valid 'dogmas' about proteins.

The false positive rates of the MisPred routines 7-10 were calculated to be 0,153, 0,149, 0,155 and 0,151, respectively, i.e. their specificities are significantly lower than those of the MisPred tools 1-6 and 11. The relatively high false positive rate of MisPred routines 7-10 reflects the fact that the DGPI constituent of these tools overpredicts GPI-anchors. On the RESULTS page of the MisPred website users are cautioned that in the case of errors identified by MisPred tools 7-10 the false positive rate is relatively high. The specificity data of MisPred tools are summarized in Supplementary Table 1.

3.2 False negative rates and sensitivity

We have monitored the false negative rates of the various MisPred tools on datasets of artificially created erroneous sequences that violate the dogma underlying the given tool. The false negative rate (β) and sensitivity ($1-\beta$) of the MisPred tools were calculated from the equation $\beta = FN/(TP + FN)$. Since all entries in the given dataset violate the given dogma, entries detected by MisPred as suspicious are true positives (TP), whereas those not detected by MisPred are false negatives (FN).

The MisPred routine for tool 1 detected 88,2% of the erroneous entries generated from secreted proteins containing an extracellular Pfam-A domain from which the N-terminal 50 residues (that might contain secretory signal peptide) were removed.

The MisPred routine for tool 2 detected 84,8% of the erroneous entries generated from transmembrane proteins containing both an extracellular and a cytoplasmic Pfam-A domain after removing their transmembrane helices.

The sensitivity of the MisPred routine for tool 3 was found to be 99,9% when we applied it to chimeric proteins generated by fusion of human Swiss-Prot proteins containing extracellular domains to proteins containing nuclear domains.

The sensitivity of the MisPred routine for tool 4 was found to 92,5% when we applied it to proteins generated by deleting 50% of the residues from the N-terminal, C-terminal and internal regions of Pfam-A domains suitable for the detection of domain size deviation.

The sensitivity of the MisPred routine for tool 5 was found to be 92,9%, when tested on artificial chimeras generated by random fusion of genes encoded on different chromosomes.

The MisPred routine for tool 6 detected 84,6% of the erroneous entries generated from transmembrane proteins containing both a signal peptide and a cytoplasmic Pfam-A domain after removing their transmembrane helices.

The MisPred routine for tool 7 detected 89,1% of the erroneous entries generated from GPI-anchored proteins after removing their signal peptide.

The sensitivity of MisPred routines for tools 8, 9 and 10 were found to be 95,2%, 95,4% and 87,2%, respectively when tested on chimeric proteins generated by fusion of human Swiss-Prot proteins containing a GPI-anchor with entries containing cytoplasmic or nuclear domains or transmembrane helices.

The sensitivity of MisPred routine for tool 11 was found to be 86,5% when tested on erroneous entries generated by deleting (or inserting) randomly selected Pfam-A domains from or into various positions of one member of the pair of orthologous sequences. The sensitivity data of MisPred tools are summarized in Supplementary Table 1.

4. Datasets analyzed

We have analyzed protein sequences of 19 Metazoan species deposited in the UniProtKB/Swiss-Prot, UniProtKB/TrEMBL (UniProt Consortium, 2012), NCBI/RefSeq (Pruitt *et al*. 2012) and EnsEMBL (Flicek *et al*. 2012) databases.

The UniProtKB Swiss-Prot and TrEMBL entries from UniProtKB release 2012_05 (May 2012) were downloaded from http://www.uniprot.org.

The protein sequences of the EnsEMBL database were downloaded from the EnsEMBL website, release 67 (May 2012), found at ftp://ftp.ensembl.org/pub/release-67.

The NCBI/RefSeq protein sequences were downloaded from NCBI website found at http://www.ncbi.nlm.nih.gov on May 2012.

References

Bendtsen,J.D. et al. (2004) Improved prediction of signal peptides: SignalP 3.0. *J. Mol. Biol.* **340**, 783-795.

Finn,R.D. et al. (2011) HMMER web server: interactive sequence similarity searching. *Nucleic Acids Res.,* **39**(Web Server Issue), W29-W37.

Flicek,P. et al. (2012) Ensembl 2012. *Nucleic Acids Res.,* **40**(Database Issue), D84-90.

Hiller,K. et al. (2004) PrediSi: prediction of signal peptides and their cleavage positions. *Nucleic Acids Res.,* **32**(Web Server Issue), W375-9.
Käll,L. et al. (2007) Advantages of combined transmembrane topology and signal peptide prediction--the Phobius web server. *Nucleic Acids Res.,* **35**(Web Server Issue), W429-32.
Kent,W.J. (2002) BLAT–the BLAST-like alignment tool. *Genome Res.,* **12**, 656–664.
Krogh,A. et al. (2001) Predicting transmembrane protein topology with a hidden Markov model: Application to complete genomes. *J. Mol. Biol.,* **305**, 567-580.
Kronegg,J. and Buloz,D. (1999) Detection/prediction of GPI cleavage site (GPI-anchor) in a protein (DGPI). http://dgpi.pathbot.com/
Marchler-Bauer, A. et al. (2013) CDD: conserved domains and protein three-dimensional structure. *Nucleic Acids Res.* **41(D1),** D348-352.
Pruitt,K.D. et al. (2012) NCBI Reference Sequences (RefSeq): current status, new features and genome annotation policy. *Nucleic Acids Res.,* **40**(Database Issue), D130-5.
Punta,M. et al. (2012) The Pfam protein families database. *Nucleic Acids Res.*, **40**(Database Issue), D 290-301.
Tordai,H. et al. (2005) Modules, multidomain proteins and organismic complexity. *FEBS J.*, **272**, 5064-78.
UniProt Consortium (2012) Reorganizing the protein space at the Universal Protein Resource (UniProt). *Nucleic Acids Res.,* **40**(Database Issue), D71-5.

Supplementary Table 1.
Specificity and sensitivity data of MisPred tools

MisPred tool	Specificity	Sensitivity
Tool 1	≥ 0,985	0,882
Tool 2	≥ 0,985	0,848
Tool 3	≥ 0,985	0,999
Tool 4	≥ 0,985	0,925
Tool 5	≥ 0,985	0,929
Tool 6	≥ 0,985	0,846
Tool 7	0,847	0,891
Tool 8	0,851	0,952
Tool 9	0,845	0,954
Tool 10	0,849	0,872
Tool 11	≥ 0,985	0,865
